# A strategic thinking of the scientific system of drug regulatory in China - advancing drug standards of China

**DOI:** 10.1186/1479-5876-10-S2-A39

**Published:** 2012-10-17

**Authors:** Fucheng Zhou

**Affiliations:** 1 Pharmacopeia Commission of China, Beijing, China

## 

Drug is special goods to prevent diseases or used for rehabilitation and health care, and therefore is essential for human society development. Drug safety directly related to people’s health and life safety, and also tightly associated with the government management and public safety. Our national pharmaceutical and health care policy has paid equal attentions on Traditional Chinese Medicine and western chemical drugs, and implemented the significant new drugs creation strategy. Plenty of strategies, approaches and technologies have been applied in the drug research field, and have promoted the fast development of biomedical industry. Now we are on the way to be a big country of pharmacy, which supplies favorable conditions and available development spaces of constructing the novel drug regulatory system.

According to the overall serious problems of China biomedical industry, such as small scale, low concentration; excess capacity, weak innovation and competition on market, and homogenization under low-end technology and quality, etc. Additionally, the development of society is facing urbanization, industrialization and aging problems, new requirements of the safety, efficient and availableness of drug have been raised. Therefore, Constructing the new system of national drug regulatory, improve the level of drug quality control and ensure the drug safety in society are essential products as the progress of times and society development, and also are important questions that to pay more attentions during drug regulatory process. We need to deeply understand them with the basis of practice and innovate drug concept and measures, to ensure the drug safety and efficient is controllable. After all, we need to persist on thinking systematically, enhance the top design, emphasize on the entire process regulatory, to construct a new system of drug regulatory which is suitable to domestic conditions.

